# In Vivo Metabolic Responses to Different Formulations of Amino Acid Mixtures for the Treatment of Phenylketonuria (PKU)

**DOI:** 10.3390/ijms23042227

**Published:** 2022-02-17

**Authors:** Nadia Giarratana, Luciana Giardino, Andrea Bighinati, Giorgio Reiner, Júlio César Rocha

**Affiliations:** 1 APR Applied Pharma Research SA, 6828 Balerna, Switzerland; giorgio.reiner@apr.ch; 2 DIMEVET—Department of Veterinary Medical Sciences, Bologna University, 40126 Bologna, Italy; luciana.giardino@unibo.it (L.G.); andrea.bighinati@unimore.it (A.B.); 3 Nutrition and Metabolism, NOVA Medical School, Faculdade de Ciências Médicas, Universidade NOVA de Lisboa, 1169-056 Lisboa, Portugal; rochajc@nms.unl.pt; 4 Reference Centre of Inherited Metabolic Diseases, Centro Hospitalar Universitário de Lisboa Central, Rua Jacinta Marto, 1169-045 Lisboa, Portugal; 5 CINTESIS—Center for Health Technology and Services Research, NOVA Medical School, Campo dos Mártires da Pátria 130, 1169-056 Lisboa, Portugal

**Keywords:** phenylketonuria (PKU), amino acid mixtures, metabolic control, BUN, prolonged release, catabolism, oxidation

## Abstract

Phenylketonuria (PKU) is a rare autosomal recessive inborn error of metabolism where the mainstay of treatment is a Phe restricted diet consisting of a combination of limited amounts of natural protein with supplementation of Phe-free or low-Phe protein substitutes and special low protein foods. Suboptimal outcomes may be related to the different absorption kinetics of free AAs, which have lower biological efficacy than natural proteins. Physiomimic Technology^TM^ is a technology engineered to prolong AA (AA-PT) release allowing physiological absorption and masking the odor and taste of free AAs. The aim of these studies was to assess the impact of AA-PT formulation on selected functional and metabolic parameters both in acute and long-term experimental studies. Adult rats in fasting conditions were randomized in different groups and treated by oral gavage. Acute AA-PT administration resulted in significantly lower BUN at 90 min versus baseline. Both BUN and glycemia were modulated in the same direction as intact casein protein. Long-term treatment with AA-PT significantly reduces the protein expression of the muscle degradation marker Bnip3L (−46%) while significantly increasing the proliferation of market myostatin (+58%). Animals dosed for 15 days with AA-PT had significantly stronger grip strength (+30%) versus baseline. In conclusion, the results suggest that the AA-PT formulation may have beneficial effects on both AA oxidation and catabolism with a direct impact on muscle as well as on other metabolic pathways.

## 1. Introduction

Phenylketonuria (PKU) is a rare autosomal recessive inborn error of metabolism where a phenylalanine hydroxylase (PAH) deficiency leads to the accumulation of amino acid phenylalanine (Phe) in the blood and brain. Untreated PKU is characterized by autism, aberrant behaviour, developmental problems, eczematous rash, irreversible intellectual disability, microcephaly, motor deficits, seizures and psychiatric symptoms [1,2]. Despite pharmacological options, a Phe restricted diet composed of a few natural proteins combined with protein substitutes, particularly Phe-free L-amino acid mixtures and special low protein foods, remains the mainstay of treatment for life [3,4]. Although improvements were observed in recent years regarding the delivery and compositions of protein substitutes, suboptimal outcomes are still reported in the literature, probably reflecting non-perfect compliance but also as a potential consequence of their chronic metabolic implications [5,6,7,8].

**Figure 1:**
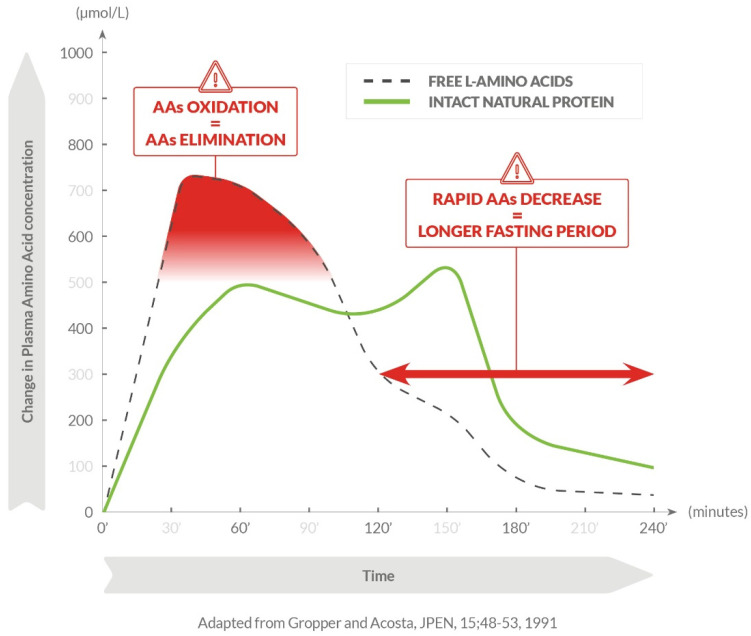
Oxidation and catabolism after ingestion of free AAs and a natural protein.

The description of such suboptimal outcomes may be related to the synthetic properties of the diet, mainly free L-amino acids which have absorption kinetics different from AAs that make up natural dietary proteins [9]. A recent meta-analysis showed that growth impairment is still a reality in some patients with PKU under a Phe-restricted diet, while the same was not found in those patients not requiring dietary restriction [8]. The different absorption kinetics of AAs from different sources may impact nitrogen balance and involve several distinct metabolic pathways [10]. Slowly digested proteins provide the efficient postprandial utilization of dietary nitrogen [11], whereas free AAs are absorbed too rapidly to support anabolic requirements [12]. Infusion of free AAs in fasted healthy adults may also result in an increase in AA oxidation [13]. In addition, prolonged release of AAs by a protein substitute may therefore be able to better support anabolic requirements and prevent or reduce catabolic episodes [9], potentially being able to minimize the impact on suboptimal outcomes in PKU patients [8] (Figure 1).

Blood urea nitrogen (BUN), an indicator of AA oxidation, is a useful measure of the ability to retain dietary nitrogen originating from AAs [14]. In the short-term (‘acute’) context, BUN is a metabolic marker of oxidation of supplemented AAs due to saturation of anabolic processes that incorporate supplemented AAs into nascent proteins [11,15]. Higher BUN is, therefore, a biomarker suggesting that supplemented AAs are not used for protein synthesis but are instead catabolized and utilized for energy production [14]. Overall, it is well known that dietary AA formulations consist of synthetic-free AAs, which have lower biological efficacy than natural protein [3]. This is the reason for which PKU patients are recommended an additional 20–50% of total protein intake from food plus an AA supplement compared to the daily recommended intake for healthy individuals [3,16]. The increased nitrogen intake recommended for PKU patients could be related to the high oxidation due to their rapid, non-physiological absorption causing an inefficient absorption/utilization rather than having better metabolic control [3,6]. However, in chronic conditions, during the fasting state, BUN is a marker of proteolysis initiated in order to release AAs from tissue for the maintenance of plasma AA homeostasis [11]. The muscle is the primary organ affected by catabolic episodes in PKU patients through proteolysis [3]. Nowadays, there are different protein substitutes, but no one of them is able to overcome the above-described differences between L-amino acid mixtures and natural protein, and there is a constant need to further improve the metabolic impact of protein substitutes [5]. Protein substitutes with the ability to prolong absorption of AAs, mimicking physiological absorption kinetics of intact natural proteins, may allow more efficient AA utilization and thus contribute to support effectively normal growth and overall healthy body composition. Positive consequences on neurocognitive performance via a better balance in brain AA concentrations are also plausible [6]. A novel nitrogen source (i.e., AAs) for a patient with PKU was developed using Physiomimic Technology^TM^ (PT), which is a technology engineered to prolong AA release in the gut allowing a physiological absorption and mask the odour and taste of free AAs [9]. The PT AA formulation (AA-PT) reduces peak AA concentrations while maintaining similar areas under the AA concentration/time curve (AUC), keeping a significantly higher concentration of blood AAs (respect to free AAs) up to 7 h (Clast), suggestive of prolonged AA release [9,17]. A further analysis suggests that the production of urea in proportion to systemic AA availability was significantly smaller after the administration of AA-PT compared with the control increasing utilization of AAs for protein synthesis and reducing their oxidation and conversion to urea [18].

The aim of this study was to assess the impact of a prolonged release of amino acids formulation on nitrogen balance both in acute and long-term experimental studies and to evaluate the impact on selected metabolic and functional parameters relative to the natural slow-release reference protein casein or free AA controls.

## 2. Results

### 2.1. Acute Effects of AA Formulations on AA Oxidation (BUN)

**Figure 2:**
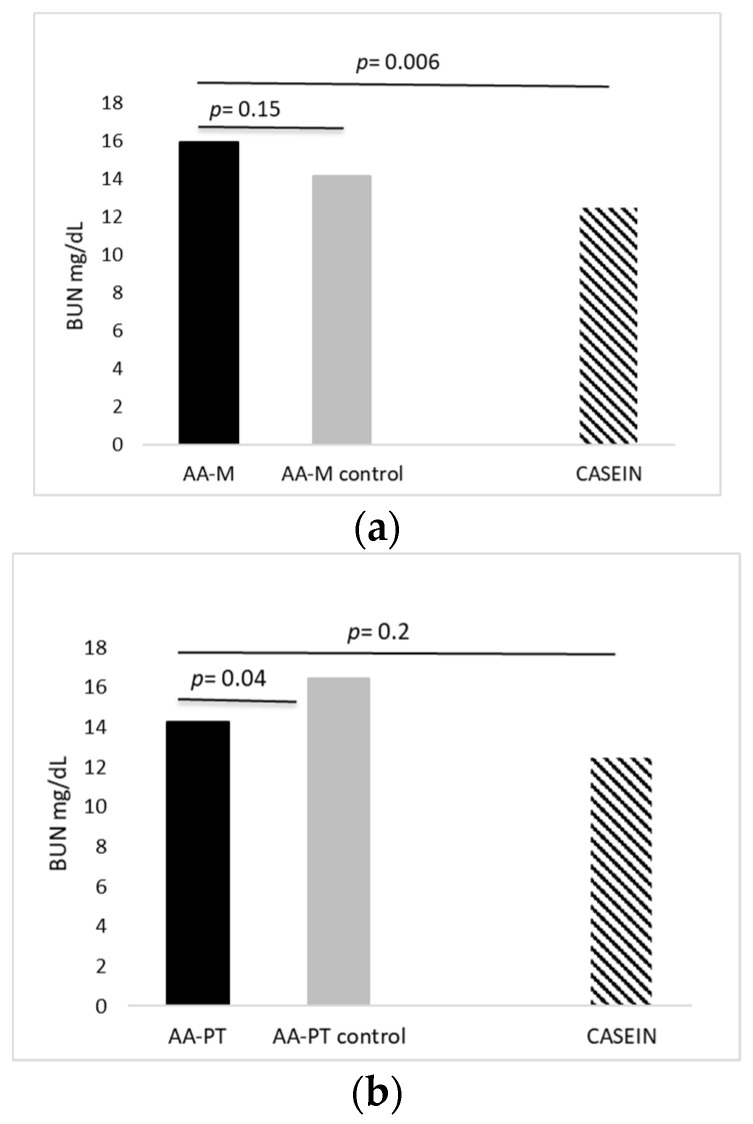
Acute effects of AA-PT, AA-M and casein on BUN. (**a**) Acute effects of AA-PT (*n* = 7; 14.27 ± 0.83 mg/dL), AA-PT-C (*n* = 7; 16.41 ± 0.46 mg/dL) and casein (*n* = 12; 12.45 ± 0.84 mg/dL) on BUN. (**b**) Acute effects of AA-M (*n* = 11; 15.95 ± 0.75 mg/dL), AA-M-C (*n* = 10; 14.17 ± 0.92 mg/dL) and casein (*n* = 12; 12.45 ± 0.84 mg/dL) on BUN. Unpaired *t*-test.

Acute AA-PT administration resulted in significantly lower BUN at 90 min compared to AA-PT-C (14.27 ± 0.83 mg/dL vs. 16.41 ± 0.46 mg/dL; *p* = 0.04) but not different (i.e., similar) when compared with intact casein (14.27 ± 0.83 mg/dL vs. 12.45 ± 0.84 mg/dL; *p* = 0.171; Figure 2a). Acute AA-M administration resulted in unchanged BUN at 90 min compared to AA-M-C (15.95 ± 0.75 mg/dL vs. 14.17 ± 0.92 mg/dL; *p* = 0.149) and significantly higher BUN compared to natural protein casein (15.95 ± 0.75 mg/dL vs. 12.45 ± 0.84 mg/dL; *p* = 0.006; Figure 2b).

### 2.2. Acute Effects on Blood Glucose and Other Biomarkers by AA Formulations and Casein

**Figure 3:**
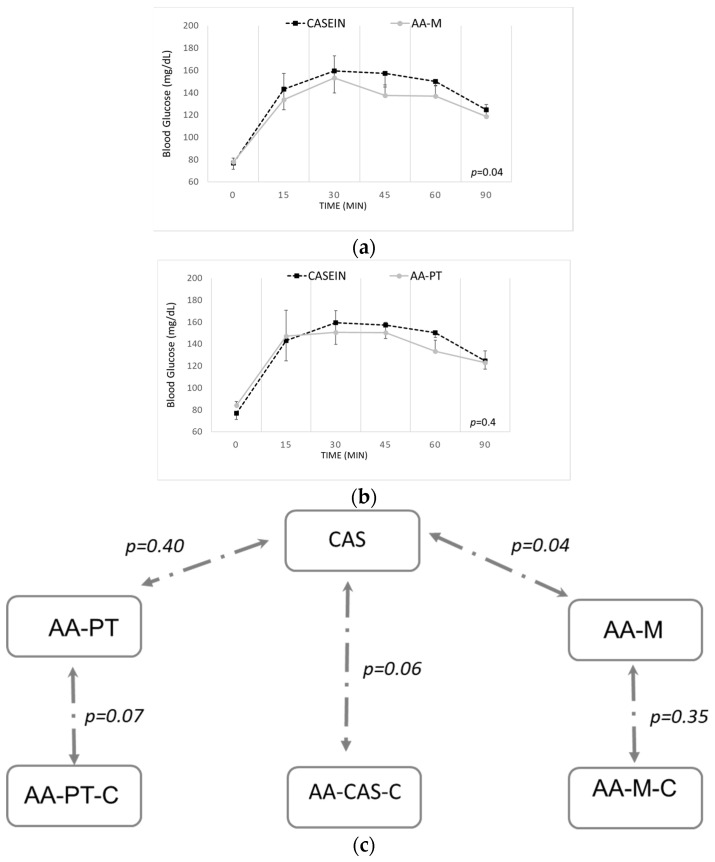
Acute effects on blood glucose by casein and the AA-PT and AA-M formulations. (**a**) AA-PT vs. casein. AA-PT: *n* = 10 animals; casein: *n* = 12 animals. *p* = 0.40; (**b**) AA-M vs. casein. AA-M: *n* = 11 animals; casein: *n* = 12 animals. *p* = 0.04. (**c**) *p*-values for the differences between glycaemic trends (two-ways ANOVA).

The glycemia trend with AA-PT, engineered with the Physiomimic Technology^TM^, did not differ significantly from that obtained with intact slow protein casein (*p* = 0.40; Figure 3a). Differently, the trend with AA-M, AAs in the form of microtabs, was significantly different from intact casein (*p* = 0.04; Figure 3b). While the glycaemic trend with AA-M did not differ significantly from that of its control AA-M-C, the glycemia trend in rats fed AA-PT vs. its control (AA-PT-C) seemed to go in the same direction as the glycemia trend of rats fed casein vs. its control (Figure 3c). No significant impact on CRP, GIP, GLP-1, insulin, ghrelin or glucagon was detected following the acute administration of any of the tested AA formulations compared with the casein (data not shown).

### 2.3. Long Term Effect of AA Formulations on Muscle

Animals lost some weight due to the gavage and the different feeding pattern than their normality due to the experimental conditions. The weight trends were very similar in the two groups: from 206 to 187 g in the AA-PT group; from 205 to 189 g in the AA-PT-C group.

**Figure 4:**
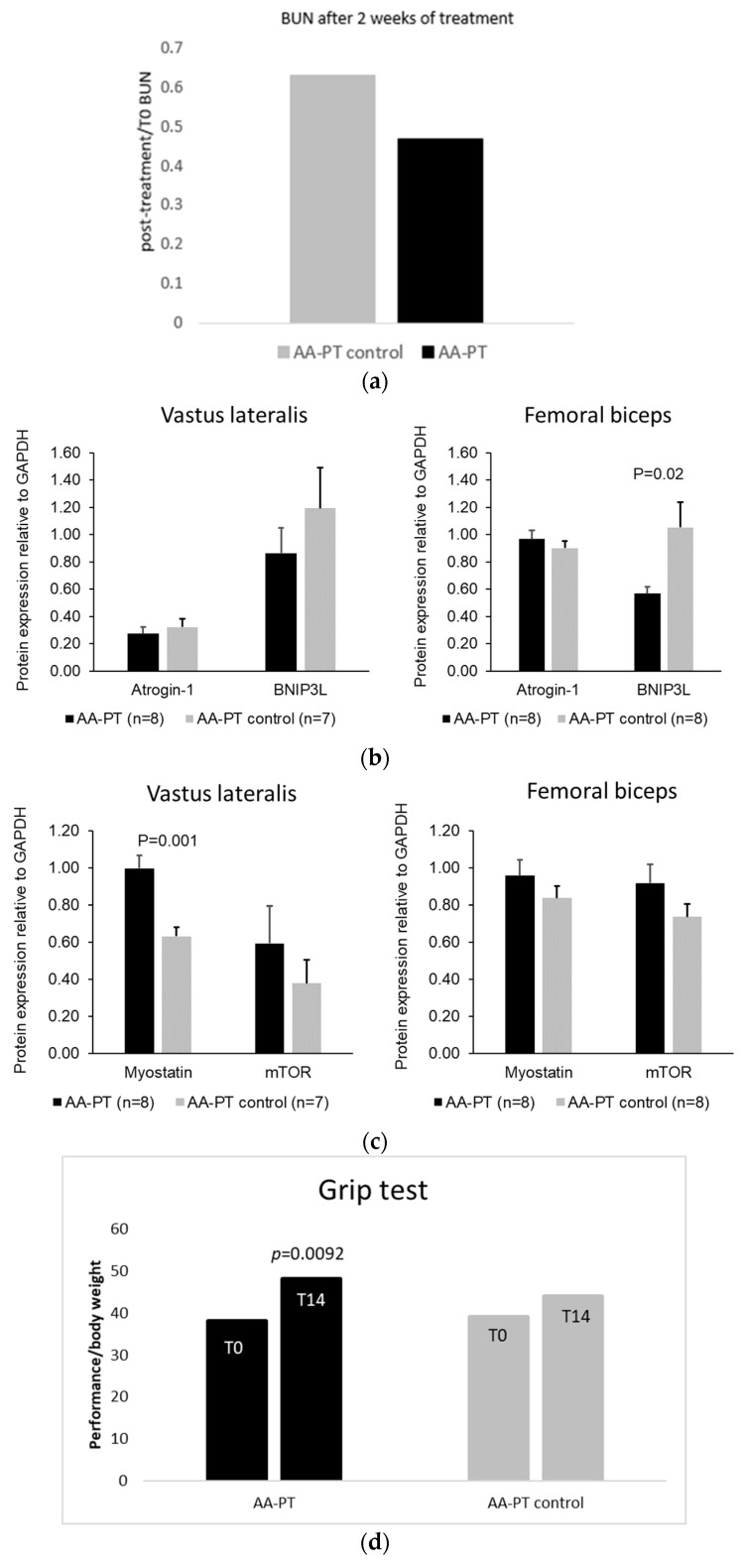
Long-term (2 weeks) effects of AA-PT and AA-PT-C on protein catabolism and protein markers of the degradation and proliferation of muscle tissue and muscle strength. (**a**) Effects of AA-PT and AA-PT-C, respectively, on relative BUN (normalized to baseline BUN for each animal) (*n* = 5 animals; 0.63 ± 0.24 vs. 0.47 ± 0.28; *p* = 0.37). (**b**) Effect of AA-PT and AA-PT-C on the relative expression of the muscle degradation markers Atrogin-1 and Bnip3L in rat vastus lateralis and femoral biceps biopsies (*n* = 8 AA-PT and *n* = 7 AA-PT-C animals). (**c**) Effect of AA-PT and AA-PT-C on the relative expression of the muscle synthesis markers myostatin and mTOR in rat vastus lateralis and femoral biceps biopsies (*n* = 8 AA-PT and *n* = 7 AA-PT-C animals). (**d**) Effects of long-term administration of AA-PT and AA-PT-C on muscle strength. Relative muscle strength is reported as grip strength (g) normalized to body weight (g) (*n* = 8 animals).

Long-term dosing of animals with AA-PT resulted in lower BUN production compared with animals given the relative amino acids without the Physiomimic Technology^TM^ (AA-PT-C) (both data normalized to baseline) (0.63 ± 0.24 vs. 0.47 ± 0.28, *p* = 0.37) (Figure 4a).

The relative protein expression of the muscle degradation marker Bnip3L was expressed significantly lower relative to Gapdh (−46%; 0.57 ± 0.15 vs. 1.05 ± 0.52; *p* = 0.02) in femoral biceps muscle of animals treated with AA-PT compared with AA-PT-C treated animals (Figure 4b). A similar, but non-significant, trend was observed in the vastus lateralis muscle (0.86 ± 0.52 vs. 1.19 ± 0.79; *p* = 0.35).

Conversely, protein myostatin was expressed significantly higher relative to Gapdh (+58%; 1.00 ± 0.20 vs. 0.63 ± 0.13; *p* = 0.001) in vastus lateralis muscle of animals treated with AA-PT compared with AA-PT-C treated animals (Figure 4c). A similar but non-significant trend was observed in the femoral biceps muscle: (0.96 ± 0.24 vs. 0.90 ± 0.24; *p* = 0.65).

The clinical impact of catabolic episodes was evaluated on in vivo muscle as grip strength (expressed in grams) normalized to total body weight at baseline and after long-term administration of either AA-PT or AA-PT-C. Animals dosed with AA-PT had significantly stronger grip strength relative to the baseline (+30%; 38.11 ± 7.47 vs. 48.18 ± 5.76; *p* = 0.009), whereas no significant increase in muscle strength was observed in animals treated with AA-PT-C (+13%; 39.21 ± 5.43 vs. 44.09 ± 5.47; *p* = 0.510; Figure 4d).

### 2.4. Long-Term Effects of the Different AA Formulations on Glucose Tolerance

**Figure 5:**
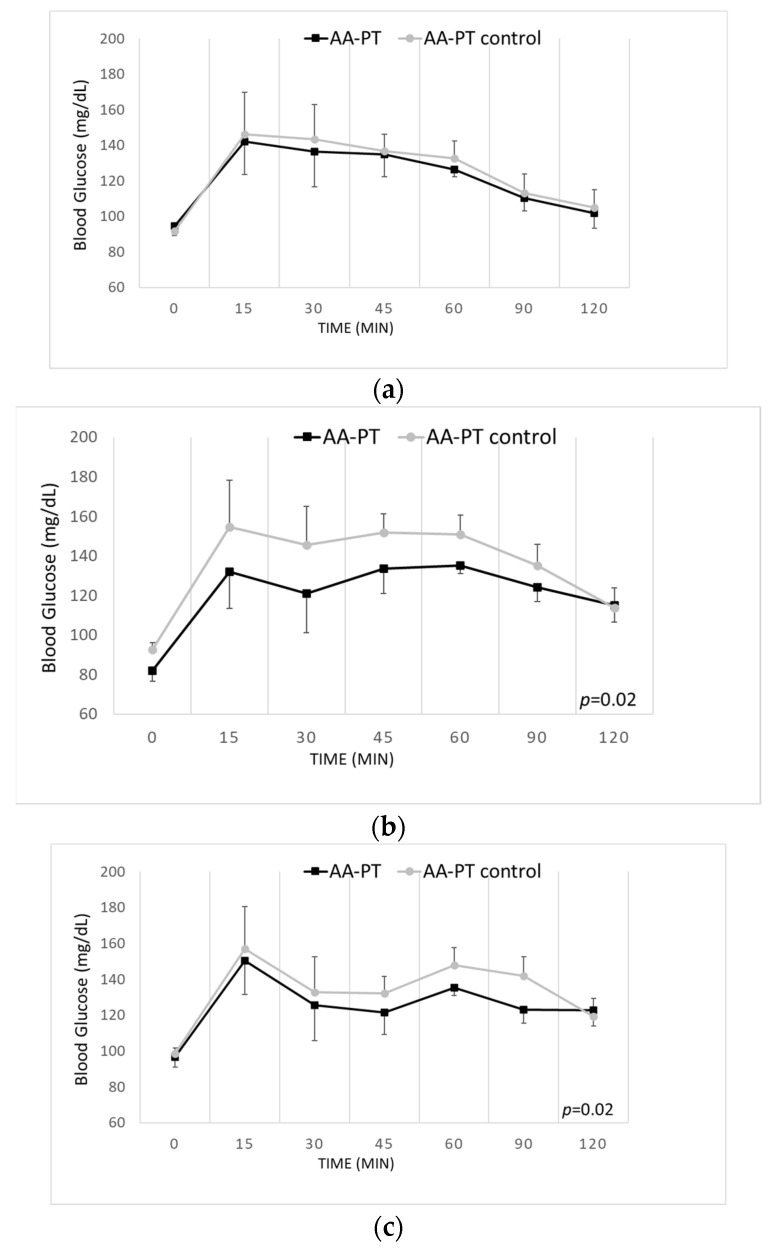
Long-term (2 weeks) effects of AA-PT versus AA-PT-C on glucose tolerance. (**a**) Blood glucose at glucose tolerance test at day 2 (*n* = 5 AA-PT and AA-PT-C); (**b**) Blood glucose at glucose tolerance test at day 7 (*n* = 4 AA-PT and *n* = 5 AA-PT-C); (**c**) Blood glucose at glucose tolerance test at day 14 (*n* = 5 AA-PT and *n* = 5 AA-PT-C).

Glucose tolerance was evaluated on days 2, day 7 and day 13 of either AA-PT or AA-PT-C dosing. Although no differences were observed on day 2 (*p* = 0.33; Figure 5a), the glucose curves obtained in AA-PT dosed animals differed significantly from the glucose curves obtained in the AA-PT-C group at days 7 and 14 (*p* = 0.02 and *p* = 0.02, respectively; Figure 5b,c).

## 3. Discussion

These in vivo animal studies explored the effects of acute and long-term ingestion of delayed amino acids on nitrogen balance evaluated as AA oxidation, protein catabolism, markers of muscle turnover and grip strength and also the impact on other metabolic pathways such as glucose metabolism.

AAs in the AA-PT formulation, which were engineered with Physiomimic Technology^TM^, are absorbed differently from free AAs, in a way more similar to natural proteins [9,17]. In addition to favoring a positive nitrogen balance [18], more physiological absorption of AAs in the dietary treatment of PKU may be expected to modulate several metabolic markers such as glycaemia [6]. The excipients of Physiomimic Technology (alginates and ethilcellulose) are commonly used in the food industry and considered safe for children, during pregnancy and breastfeeding.

The physiological production of urea reflects the pattern of utilization of ingested AA in the body [19]. Higher BUN and subsequent urinary nitrogen excretion suggest that AAs are not preferentially targeted to protein synthesis but are instead oxidized for energy production [20]. We measured the effect of acute administration of different AA formulations (AA-PT and AA-M) and a slow natural protein as casein on BUN, which represents a well known metabolite of AA oxidation. Results showed that, relative to their respective controls, AA-PT (with delayed release AAs) significantly produced lower BUN. Differently, AA-M formulation had no effect on BUN with respect to its control AA-M-C. These observations are in line with the concept that the kinetic of AAs absorption is important to determine the biological value of the ingested nitrogen source resulting in an increased amino acid intake as suggested by the PKU guidelines [3,16]. AA mixture engineered to prolong AA absorption with Physiomimic Technology^TM^ dosed as the AA-PT formulation may be less oxidized than the correspondent control of AAs in the same direction as natural protein casein. On the contrary, the microtabs (AA-M) formulation was not able to reach the same result being essentially similar to the correspondent free AAs control, without any apparent impact on nitrogen metabolism.

The nitrogen balance is not only important for the metabolism of an external source of AAs or protein ingested but also at chronic levels to keep a good catabolic/anabolic balance in the physiological conditions [11,20]. In long-term dosing experiments, two weeks of dosing with the AA-PT formulation resulted in reduced BUN compared to the control formulation (AA-PT-C), which suggests that long term treatment with prolonged release AAs results in less protein (i.e., muscle) catabolism compared with administration of relative free AAs with fast absorption kinetic. This conclusion was further supported by Western Blot experiments demonstrating reduced relative expression of the muscle degradation marker Bnip3L and approximately 50% increased relative expression of the muscle proliferation marker myostatin in vastus lateralis and femoral biceps muscle biopsies, respectively. The slight differences in relative expression levels seen in the two samples muscle types may be due to differences in the proportion of type I and type II muscle fibers in the two muscles, as type I and type II muscle fibers may express slightly different levels of Bnip3L, myostatin or the reference protein Gapdh [21]. The positive effect on muscle mass was further investigated in grip strength tests, which demonstrated that long-term dosing with AA-PT significantly increased muscle strength relative to baseline. The importance of avoiding muscle degradation is highlighted by the fact that endogenous muscle proteins contain substantial amounts of Phe, which are released into the circulation when muscle proteins are degraded and may therefore aggravate signs and symptoms of PKU in spite of adherence to a low Phe diet [22], with a reported increase of Phe levels in the early morning after a long fasting period (night) [23].

Indeed, most amino acids have important functions in metabolic health outside their role in protein synthesis [24]. Human studies have demonstrated that fast absorbable proteins elicit greater insulin responses than slow proteins, which consequently translates to a stronger lowering of blood glucose levels [25]. In our experiments, a significant reduction in blood glucose was seen following acute administration of AAs in AA-M formulation (i.e., microtabs), but not with intact casein or AAs in AA-PT (i.e., engineered with Physiomimic Technology^TM^) formulation. Considering that nutrients’ profile of AA-M and AA-PT are similar, this result suggests that AAs delivered either as part of an intact protein (i.e., casein) or as the AA-PT formulation may have delayed absorption in vivo and a less pronounced effect on insulin release and blood glucose levels. These preliminary results could be clinically promising, as PKU patients are at high risk of carbohydrate intolerance and insulin resistance due to their high caloric intake in the form of carbohydrates [26] and glycemic index of the special low protein foods. AA formulations able to mitigate this increased risk of metabolic disease would therefore be advantageous. The less pronounced insulin and glucose excursions observed with AA-PT products may help to maintain a normal satiety response, but this needs to be further studied. The findings from this single-dose study may be predictive of sustained health benefits if a prolonged-release product is consumed regularly [17]. As several factors are involved in these processes, this is promising information needs to be further investigated and confirmed.

Limitations of the study include the in vivo animal study design, gavage administration of the investigated formulations, relatively small numbers in each treatment group and the short treatment period of up to two weeks. Healthy rats were used assuming that the metabolism of PKU patients is the same as healthy people [27,28]. Additionally, the plasma amino acid concentrations were not measured after feeding, assuming that differences could be related to the prolonged release effect as previously demonstrated [9,17]. Strengths of the study include the mutually supporting experimental outcomes on glucose tolerance, BUN and muscle mass and grip strength, which taken together, support the notion that the AA-PT formulation more closely mimics the biochemical properties of intact proteins than the other investigated AA formulations.

In conclusion, the results of these in vivo studies suggest that AA-PT formulation may have beneficial effects on both AA oxidation and catabolism with a direct impact on muscle mass and muscle strength as well as on other metabolic pathways such as glycaemia in normal rats. The direct comparison between AA-PT and AA-PT-C allows us to demonstrate that the metabolic benefits are derived only from the different absorption kinetic of the same mix of amino acids and is not related to the amino acid content itself. The results of this study also support further studies in human subjects aimed at demonstrating the beneficial effects of the Physiomimic Technology^TM^ AA formulation for the dietary treatment of PKU.

## 4. Materials and Methods

### 4.1. Animals and Experimental Procedures

Both the acute and the long-term dosing experiments were performed in adult male Wistar rats (Charles River Laboratories Italia, Calco, Italy) with a weight range of 180–337 g. Animals were randomized in treatment groups based on the experimental design.

Animals received the various AA treatments by means of oral gavage performed with plastic flexible tubes, with the atraumatic tip of the correct length to cover the distance from the tip of the nose to the xyloid process. The probe was attached to a 2.5 mL syringe containing the suspension or solution to be delivered slowly to the animal under light anesthesia with isoflurane. Good practice standard procedures [29] were used for containing the animal in a vertical position, and the probe was slid into the esophagus. After administration of the compound at a controlled speed, the animals were returned to their cages and monitored for signs of respiratory distress or regurgitation. The number of animals per group varied according to the operational procedures and related problems (i.e., gavage) or deviation from the protocol.

### 4.2. Acute Experiment 

Animals were fasted for 12 h prior to gavage, and water was forbidden from one hour before treatment administration until the end of the experiment. The animals were sacrificed 90 min post gavage, and blood samples were collected and stored for biomarker analysis.

Two different AA delivery formulations (AA-PT [Physiomimic Technology^TM^ AAs by Applied Pharma Research s.a]; and AA-M [microtabs formulation AAs from the market]) were assessed in comparison to casein, a natural slow-release protein. Since each of the three tested formulations has a unique AA composition, a matched control mix of free AAs was prepared as a control for each experimental group (AA-PT-C, AA-M-C and AA-casein-C, respectively). Additional nutrients were also added to the AA-PT-C and AA-M-C formulations in order to mimic the composition of the AA-PT and AA-M formulations. Each animal was dosed with the equivalent of 0.7 g AAs per kg body weight in a solution of 20% glucose (8 mL/kg body weight). The animals were randomized into treatment groups 6–12 animals/group.

#### Acute Metabolic Marker Analysis

Blood glucose was sampled using a small amount of blood from the tail using Ascensia Contour Next strips and a Bayer Contour X glucose meter (Ascensia Diabetes Care-US) 10 min pre gavage and 15, 30-, 45-, 60- and 90 min post gavage. Only animals with fasting blood glucose within the normal range (i.e., ≤136 mg/dL) [30] were included in the analysis. The animals were sacrificed 90 min post gavage. A panel of rat metabolic biomarkers (insulin, total gastric inhibitory polypeptide (GIP), active glucagon-like peptide-1 (GLP-1), glucagon, ghrelin; by Merch Millipore) was analyzed using xMAP multiparametric technology on a MagPix instrument following the manufacturer’s instructions. C-reactive protein (CRP; by Cloud Clone Corp., Katy, TX, USA) was analyzed by enzyme-linked immunosorbent assay (ELISA). Blood urea nitrogen (BUN; SressMarq Biosciences, Inc., Victoria, BC, Canada) was quantified by quantitative colorimetric assays (Ying et al., 2016) [30].

### 4.3. Long-Term Experiment

After 2 weeks of adaptation (reduced daily protein amount of 5%), rats were administered two gavages per day with AA-PT and AA-PT-C for 2 weeks. Each gavage consisted of the equivalent of a protein dose of 1.25 g/kg bodyweight protein plus a feed including all the nutrients of a normal rat diet, except for AAs or protein. The animals were fasted, with no access to water, for two hours before and after each treatment. Other nutrients (i.e., glucose 5%, starch 5%) were dissolved in the liquid medium (i.e., water) and used for each gavage in order to mimic a complete meal. Considering that healthy rats were used for the experiments, both the AA-PT and AA-PT-C formulations were supplemented with free Phe to guarantee a physiological diet. Rats resulted to eat the same quantities of feed during the study period so feeding conditions can be considered similar (11.7 vs. 11.5 average fed gr/rat). Two rounds of experiments were performed, with 3–5 animals per group.

#### 4.3.1. Grip Test 

Muscle strength was measured 3 days before and 15 days after initiation of the gavage feeding regimen, using a computerized grip strength meter (Model 47200, Ugo-Basile, Varese, Italy). The apparatus consisted of a T-shaped metal bar connected to a force transducer set to measure a maximum force (F) of 1500 g. To measure grip strength in the forepaws of the rats, the animals were gently held at the base of the tail, allowing the rat to grasp the metal bar with its forepaws. As soon as the rats grasped the transducer metal bar with their forepaws, the experimenter pulled the animals backwards by the tail until the metal bar was released. The peak force of each measurement was automatically recorded by the device. Forelimb grip strength in each animal was measured in triplicate and normalized to body weight [31].

#### 4.3.2. Semi-Quantitative Western Blot of Muscle Protein Expression

The direct involvement of muscle in catabolic episodes was evaluated with specific markers: Atrogin-1 is implicated in ubiquitin-mediated proteolysis and muscle atrophy [32] and Bnip3L -like (also known as NIX) in muscle cell death and autophagy [33]. Conversely, Myostatin, as a differentiation/proliferation controller of myoblast [34], and mTOR as a positive regulator [35], was tested for muscle synthesis markers.

The protein expression of Bnip3L, mTOR, myostatin and atrogin-1 in vastus lateralis and femoral biceps muscle tissue was quantified after long term treatment with AA-PT and AA-PT-C by semi-quantitative Western blot (WB) using GAPDH as a reference protein, as described elsewhere [36,37]. Anti- Bnip3L primary antibodies were from Proteintech, anti-mTOR from Cell Signaling Technology, anti-myostatin from Abcam, anti-atrogin-1 from ECM Biosciences and anti-GAPDH from Genetex.

#### 4.3.3. Glucose Tolerance Test

Glucose tolerance tests were conducted at Days 0, 7 and at the end of the long-term experiment as described below. After 3 h of fasting conditions, controlled amounts of glucose (3 g/kg) were given at the time of the second round of treatment of the day instead of the AA treatment itself, and blood glucose was measured just before the glucose load and every 30 min after the load, until 120 min.

### 4.4. Statistical Analysis

The results are presented as mean + standard error of the mean (SEM). Student’s *t*-test, one-way ANOVA and post hoc tests were used to compare the effects of the different treatments.
